# Survey dataset on fusing RFID with mobile technology for efficient safety of construction professionals

**DOI:** 10.1016/j.dib.2019.104290

**Published:** 2019-08-06

**Authors:** Temidayo O. Osunsanmi, Ayodeji E. Oke, Clinton O. Aigbavboa

**Affiliations:** SARChI in Sustainable Construction Management and Leadership in the Built Environment, Faculty of Engineering and the Built Environment, University of Johannesburg, South Africa

**Keywords:** Fourth industrial revolution, Health hazards, Occupational health and safety, Radio frequency identification (RFID)

## Abstract

The fourth industrial revolution has encouraged technologies such as the RFID fused with mobile software for monitoring construction workers on site. In this dataset, a structured questionnaire was design directed to thirty-four (34) construction professionals in Gauteng province South Africa through random sampling. The set of descriptive statistics is presented with tables, bar and pie charts. The willingness level of construction professionals to adopt RFID and mobile technology on construction sites was identified. The barriers to the adoption of fusing mobile technology and RFID for construction safety can be determined when the data is analyzed. Moreover, the construction professional's agreement with RFID as a tool for preventing health hazards on construction sites can be obtained from the analysis of the survey data.

**Table:** Specifications Table

Subject area	*Construction*
More specific subject area	*Construction health and safety*
Type of data	*Table, figures and text file,*
How data was acquired	*Field Survey*
Data format	*Raw*
Experimental factors	*Random sam*pling of construction professionals comprising of quantity surveyors, architects, urban and regional planners, civil, mechanical and electrical engineers.
Experimental features	*Descri*ptive statistics of the construction professionals willingness to adopt RFID and mobile technologies on-site and barriers to the adoption of fusing mobile technology and RFID for construction safety.
Data source location	*Johannesburg, Gauteng province South Africa.*
Data accessibility	*The data are in this data article.*
Related research article	*It is a direct submission to* Data in Brief*, the most relevant research article is*[1]

**Table:** 

**Value of the Data** • The dataset provided showed the possibility for improving the safety of construction workers through effective monitoring of their activities through the use of technologies. • The data set is valuable in improving the understanding of factors that can hinder the adoption of RFID, mobile technology and other modern technologies that support monitoring of construction workers on site. • The data provide empirical evidence on the state awareness level of RFID and mobile technology on construction sites. • The dataset presents the construction professional's agreement with RFID as a tool for preventing health hazards on construction sites • The research instrument (questionnaire) utilized for generating the data set can also be used for further research by other researchers. • The data set revealed that innovative ideas for ensuring construction workers safety will require an interdisciplinary collaboration between a computer scientist and safety personnel.

## 1 Data

The data set contains responses obtained from a questionnaire survey of construction professionals ranging from quantity surveyors, architects, urban and regional planners, civil, mechanical and electrical engineers. Table 1 presents the barriers to the adoption of RFID and mobile technology for enhanced safety of construction workers. The barriers include; cost of implementation (4.53), Low technical know-how (4.50), Data security (4.29), Communication range (4.24), Storage of data (4.12), Additional weight of sensor on the PPE (4.06), Ethical considerations (3.71) and Power availability (3.62).

**Table 1:** Barriers to the use of RFID and mobile technology for construction safety.

	N	Minimum	Maximum	Mean	Rank	Std. Deviation	Cronbach AlPha
Cost of Implementation	34	3	5	4.53	1	.662	0.856
Low technical know how	34	3	5	4.50	2	.663	
Security of data	34	3	5	4.29	3	.760	
Communication range	34	3	5	4.24	4	.741	
Storage of data	34	3	5	4.12	5	.729	
Additional weight of sensor on the PPE	34	2	5	4.06	6	.776	
ethical considerations	34	2	5	3.71	7	.799	
Power availability	34	2	5	3.62	8	.985	

**Fig. 1:**
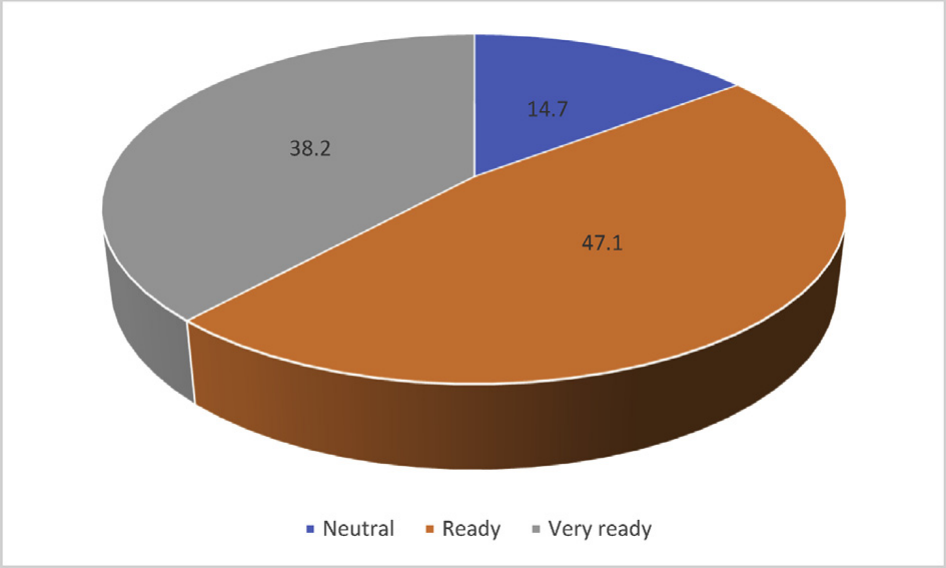
Readiness of construction professionals to adopt RFID and mobile technology.

Fig. 1 presents the readiness of construction professionals to adopt RFID and mobile technology for enhanced safety on construction sites. The chat shows that almost half (47.1) of the construction professionals are ready to adopt RFID and mobile phones. Whereas 14.7% are neutral about the adoption of RFID and 38.2% are very ready to adopt RFID and mobile technology.

**Fig. 2:**
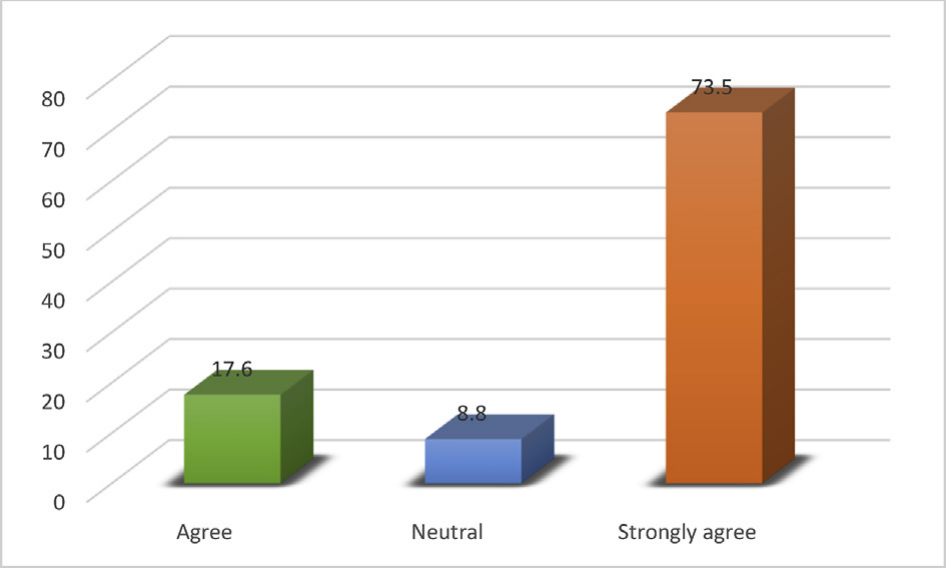
Construction professionals level of agreement with the use of RFID for enhancing safety on site.

Fig. 2 revealed the construction professionals level of agreement with RIFD as a tool for preventing occupational hazards on construction sites. Almost all (73.5%) of the respondents agreed with the opinion that RFID has the potentials for curbing the health hazards experience on construction site while 8.8% are neutral about the use of RFID.

## 2 Experimental design, materials, and methods

The weak monitoring of construction professionals activities on construction sites has been responsible for the occupational hazards of construction workers on site [2]. Towards enhancing the safety of construction professionals an experiment was conducted using radio frequency identification (RFID) and mobile technology. Radio frequency identification contains three major components which are; tag, reader and back end system [3]. The experiment proposed the fixing of the tag on the protective equipment (overall, hard hats and many other) worn by the construction professionals on the site. The safety officer's mobile phone on site will function as the RFID reader. The experiment adopted mobile technology because it is expected to provide a fast and easy way for monitoring construction workers activity remotely on site.

The data generated from the experiment was acquired through random sampling of construction professionals in Gauteng province South Africa. Past researchers [4], [5], [6] adopted a similar approach to obtain empirical data from respondents. The data was collected through a structured close-ended questionnaire directed at construction professionals (quantity surveyors, architects, urban and regional planners, civil, mechanical and electrical engineers). The questionnaire was broken down into three sections which are personal information of the respondents, readiness to adopt RFID, agreement with RFID as a tool for preventing hazards and barriers to the use of RFID and mobile technology for construction safety. The barriers to the use of RFID was acquired using a five-point Likert scale from not agree denoted by 1 to very agree represented by 5. A total of 40 construction professionals were selected with the selection based on their involvement of health and safety on the construction site. Out of the selected construction professionals, 34 responded effectively and their response was analyzed. The dataset was analyzed using SPSS and MicrosoftExcel to produce descriptive data.
